# Generation of Porcine Induced Neural Stem Cells Using the Sendai Virus

**DOI:** 10.3389/fvets.2021.806785

**Published:** 2022-01-12

**Authors:** Warunya Chakritbudsabong, Ladawan Sariya, Phakhin Jantahiran, Nattarun Chaisilp, Somjit Chaiwattanarungruengpaisan, Ruttachuk Rungsiwiwut, Joao N. Ferreira, Sasitorn Rungarunlert

**Affiliations:** ^1^Laboratory of Cellular Biomedicine and Veterinary Medicine, Faculty of Veterinary Science, Mahidol University, Nakhon Pathom, Thailand; ^2^Department of Preclinic and Applied Animal Science, Faculty of Veterinary Science, Mahidol University, Nakhon Pathom, Thailand; ^3^The Monitoring and Surveillance Center for Zoonotic Disease in Wildlife and Exotic Animals (MoZWE), Faculty of Veterinary Science, Mahidol University, Nakhon Pathom, Thailand; ^4^Department of Anatomy, Faculty of Medicine, Srinakharinwirot University, Bangkok, Thailand; ^5^Avatar Biotechnologies for Oral Health and Healthy Longevity Research Unit, Faculty of Dentistry, Chulalongkorn University, Bangkok, Thailand; ^6^Faculty of Dentistry, National University of Singapore, Singapore, Singapore

**Keywords:** Sendai virus, cell reprogramming, porcine, neurosphere, differentiation, induced neural stem cells

## Abstract

The reprogramming of cells into induced neural stem cells (iNSCs), which are faster and safer to generate than induced pluripotent stem cells, holds tremendous promise for fundamental and frontier research, as well as personalized cell-based therapies for neurological diseases. However, reprogramming cells with viral vectors increases the risk of tumor development due to vector and transgene integration in the host cell genome. To circumvent this issue, the Sendai virus (SeV) provides an alternative integration-free reprogramming method that removes the danger of genetic alterations and enhances the prospects of iNSCs from bench to bedside. Since pigs are among the most successful large animal models in biomedical research, porcine iNSCs (piNSCs) may serve as a disease model for both veterinary and human medicine. Here, we report the successful generation of piNSC lines from pig fibroblasts by employing the SeV. These piNSCs can be expanded for up to 40 passages in a monolayer culture and produce neurospheres in a suspension culture. These piNSCs express high levels of NSC markers (PAX6, SOX2, NESTIN, and VIMENTIN) and proliferation markers (KI67) using quantitative immunostaining and western blot analysis. Furthermore, piNSCs are multipotent, as they are capable of producing neurons and glia, as demonstrated by their expressions of TUJ1, MAP2, TH, MBP, and GFAP proteins. During the reprogramming of piNSCs with the SeV, no induced pluripotent stem cells developed, and the established piNSCs did not express OCT4, NANOG, and SSEA1. Hence, the use of the SeV can reprogram porcine somatic cells without first going through an intermediate pluripotent state. Our research produced piNSCs using SeV methods in novel, easily accessible large animal cell culture models for evaluating the efficacy of iNSC-based clinical translation in human medicine. Additionally, our piNSCs are potentially applicable in disease modeling in pigs and regenerative therapies in veterinary medicine.

## Introduction

The remarkable discovery that differentiated cells can be completely reprogrammed into induced pluripotent stem cells (iPSCs) by viral-mediated transduction of exogenous transcription factors marks a significant breakthrough in regenerative medicine (1). iPSCs offer an infinite supply of differentiated cells for various purposes, including disease modeling *in vitro*, drug research, toxicity testing, and autologous cell-based therapy (2). The potential for patient-specific cells to be used in autologous cell-based treatments is intriguing due to the reduction of risk from systemic immune response and the transmission of diseases via cell and tissue transplantation from the individual. The generation of neural stem cells (NSCs) and neurons from iPSCs is one of the most clinically relevant cell types (3–5). Nevertheless, this process is complicated by several factors, including a lengthy, inefficient reprogramming and differentiation processes (6), heterogenous cell differentiation (7), the possibility of tumor development as a result of undifferentiated iPSCs surviving in the differentiated iPSC population (4), and genomic instability (8).

Alternatively, the forced expression of NSC transcription factors (9–11) or pluripotency transcription factors, encoding OCT4, SOX2, KLF4, and c-MYC (OSKM) (12–14), converts differentiated cells directly into induced neural stem cells (iNSCs) and induced neural progenitor cells (iNPCs). This approach is an appealing alternative to existing iPSC technology because it enables the production of patient-specific NSCs without passing through the pluripotent stage, thereby decreasing the tumorigenic risk (15, 16). Since the first mouse iNSCs (miNSCs) were established in 2011 (12), many studies have been published detailing the derivation of iNSCs from a variety of species, including rats (17), monkeys (18), and humans (18–20). Established iNSCs have numerous features in common with embryonic brain-derived NSCs, including morphology, self-renewal capacity, gene and protein expression profiles, epigenetic state, as well as functional multipotency *in vitro* and *in vivo* (9, 21, 22). Additionally, when iNSCs are transplanted into animal models for up to 6 months, they can alleviate disease phenotypes and prevent developing tumors, thus demonstrating their therapeutic promise for neurological disorders (23).

Although iNSCs are a feasible, effective, and autologous source for medical applications, their therapeutic potential has yet to be fully explored. Porcine iNSCs (piNSCs) may serve as a disease model for human regenerative medicine, as pigs have been established as one of the most effective large animal models in biomedical research and are often regarded as a preferable alternative to rodent models (24–26). Furthermore, preclinical evaluation of stem cell transplantation using piNSCs and their differentiation cells may be utilized to determine the safety and efficacy of iNSCs prior to human trials. Importantly, piNSCs are an appealing cell source for investigating pig disease in veterinary medicine. However, no commercially available piNSCs and their neural differentiation exist for studying pig neurological diseases, such as *Streptococcus suis* infections (27) and Japanese encephalitis (28). Until now, only one group has reported success in generating piNPCs using episomal plasmids; they demonstrated that piNPCs retain the capacity to grow for an extended period of time and differentiate efficiently into neurons *in vitro* (29).

Although previous studies have established numerous methods for directly converting somatic cells into iNSCs, most of the investigations rely on integrating viral vectors (such as lentiviral or retroviral approaches) (9, 13, 15, 30, 31). These methods may result in insertional mutagenesis and the persistence or reactivation of transgenes. Moreover, therapeutic translation of this technique will require a thorough safety evaluation of mutations that occurred during the reprogramming process, as well as a fast derivation and differentiation strategy (32). A Sendai virus (SeV) vector can overcome these issues due to its single-stranded RNA virus propagating in the cytoplasm of infected cells that neither pass through a DNA phase nor integrate into the host genome, unlike other viruses. As a result, the risks of tumorigenesis are reduced throughout the reprogramming process (33). With the SeV delivery system in kits, researchers may easily transduce the desired cells with SeV carrying OSKM for reprogramming and quickly remove SeV and transgenes via a temperature change due to its temperature sensitivity. Recently, SeV-based vectors have been widely utilized to generate human integration-free iPSCs (34–37) and have been adapted for the generation of iNSCs from human and monkey postnatal and adult fibroblasts (18). However, the generation of piNSCs using the SeV has not been explored yet.

In this investigation, we generated piNSC lines from pig fibroblasts by utilizing an integration-free SeV approach. The piNSCs displayed typical features of NSCs, such as morphology, gene expression patterns, self-renewal capacity, and differentiation potential. We anticipate that piNSCs will serve as novel, easily accessible large animal cell culture models for evaluating the efficacy of iNSC-based clinical translation. This pig model will allow us to assess the ultimate feasibility of personalized cell-based therapies. Furthermore, our piNSCs might be useful for disease modeling in pigs. As a result, this discovery is beneficial for veterinary medicine and possibly translation to human medicine.

## Materials and Methods

### Ethics Statement

The Institutional Animal Care and Use Committee at the Faculty of Veterinary Science, Mahidol University, Thailand, reviewed and approved the experimental animal used in this study (Approval ID: MUVS-2015-49).

### Cell Culture

All chemical compounds and cell culture reagents were acquired from Sigma-Aldrich (St. Louis, MO, USA) or Thermo Fisher Scientific (Waltham, MA, USA), unless otherwise specified. All cells were incubated in a humidified 5% CO_2_ incubator at 37°C.

### Generation of piNSCs

**Figure 1:**
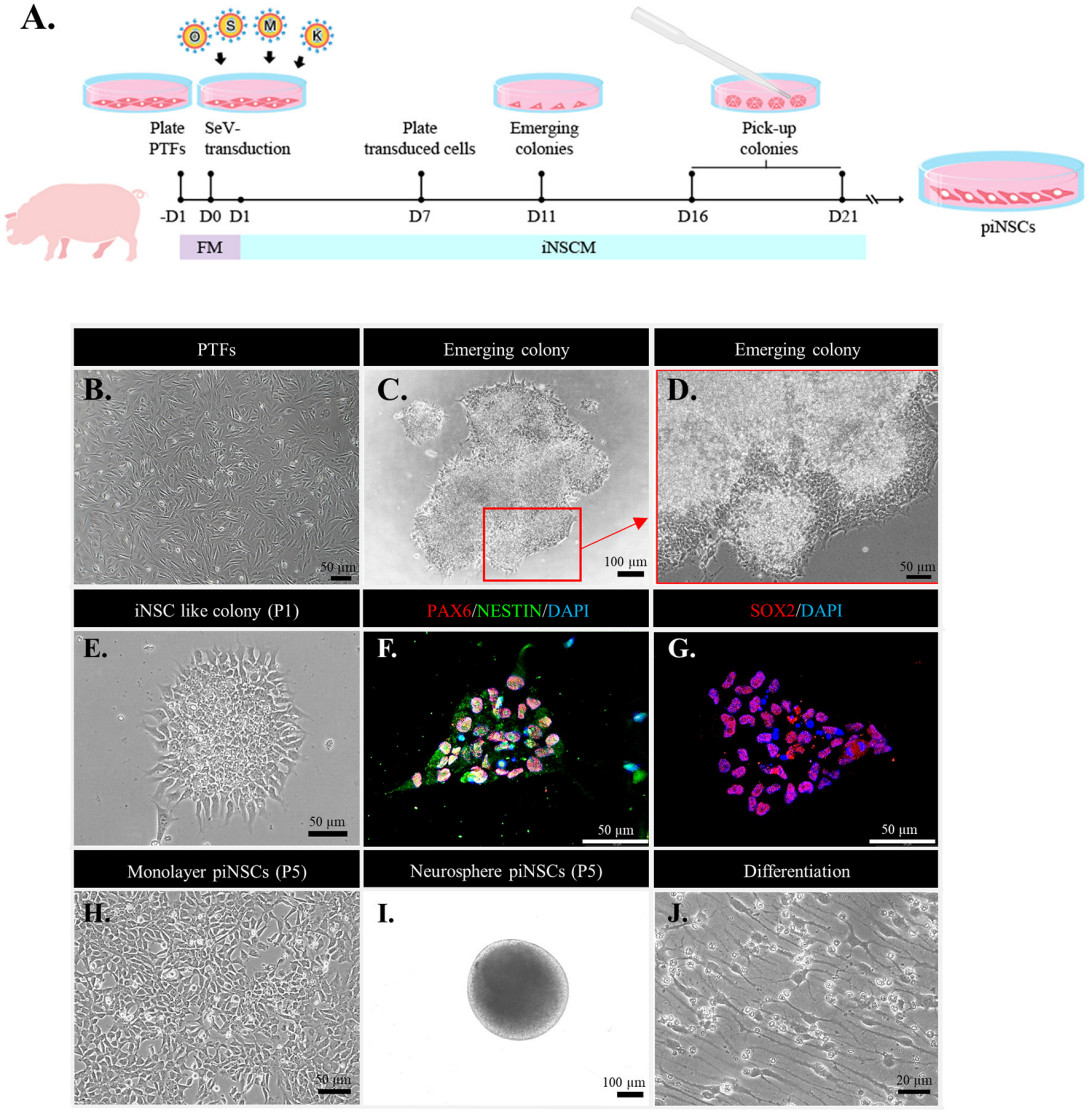
Direct reprogramming of fibroblasts into porcine induced neural stem cell (piNSC) lines using an integration-free Sendai virus (SeV). **(A)** Schematic diagram illustrating the piNSC generation timeframe. **(B)** Phase-contrast image of porcine tail fibroblasts (PTFs) treated with SeV transfection overnight. **(C)** Phase-contrast image of an emerging piNSC-like colony 21 days after transfection (Passage 0; P0). The arrow indicates the extent to which the area of the cell in **(C)** was magnified in the high-resolution image **(D)**. **(D)** High-resolution image of the cells in the inset in **(C)**. **(E)** Phase-contrast image of piNSC-like colonies after first passage (P1), which are positive for early NSC markers PAX6/NESTIN [**(F)**, red/green] and SOX2 [**(G)**, red]. DAPI staining is blue. These colonies, which were positive for PAX6/NESTIN and SOX2, would be propagated to establish self-renewing piNSC lines. Morphology of iNSCs at P5 when cultured in a Matrigel-coated dish **(H)** and the formation of neurospheres when maintained in a suspension culture **(I)**. piNSC-derived neurons showed typical neuronal morphology **(J)**.

A porcine tail was received from an authorized farm in Ratchaburi Province, Thailand. Porcine tail fibroblasts (PTFs) were extracted using standard procedures from the tail of a three-day-old crossbred piglet, with minor modifications (38). The PTFs were propagated in fibroblast medium (FM) containing DMEM-high glucose, 10% fetal bovine serum (cat. no. SV30160, Hyclone, Logan, UT, USA), 1% antibiotic-antimycotic solution, and 1% GlutaMAX™. All cells were grown on feeder-free culture systems (Matrigel-coated dishes or plates) throughout the piNSC generation process. The PTFs were reprogrammed utilizing the integration-free CytoTune™-iPS 2.0 Sendai Reprogramming Kit, carrying three vector preparations: polycistronic human KLF4-OCT3/4-SOX2 (KOS), human C-MYC, and human KLF4 (Thermo Fisher Scientific) following the manufacturer's instructions, with modifications (Figure 1A). The PTFs (passages 3) were seeded on 6-well plates at a density of 1 × 10^4^ cells/cm^2^ 1 day before viral transduction to achieve approximately 60%−70% confluency at the time of transduction. The PTFs were transfected with SeV at a multiplicity of infection of 5 in an FM for 24 h. The next day, the culture medium containing the SeV was removed and replaced with a new FM. The following day, the medium was switched to the iNSC medium (iNSCM) comprising DMEM/F-12 and a neurobasal medium at a ratio of 1:1 supplemented with 2% B-27 supplement, 1% N-2 supplement, 1% antibiotic-antimycotic solution, 1% GlutaMAX, 20 ng/mL human basic fibroblast growth factor (bFGF; R&D Systems), and 10 ng/mL human epidermal growth factor (hEGF). Seven days after reprogramming, the cells were dissociated with a 0.25% trypsin- ethylenediaminetetraacetic acid (EDTA) solution and replaced on a Matrigel-coated plate in piNSCM. Then, the appearance of epithelium-like colonies was monitored, and the medium was changed daily. Colonies with epithelium-like morphology were large enough to be collected around days 16–21 and were transferred onto an *in vitro* fertilization one-well dish for expansion. Sub-culturing at a 1:5 ratio with Versene® Solution was performed every 2–3 days for further experiments.

### Neurosphere Formation

The formation of neurospheres was investigated for iNSCs by resuspending 10,000 cells per well in iNSCM in 96-well plates covered with poly (2-hydroxyethyl methacrylate). Every 2 days, a fresh medium was added to the suspension cultures. Neurospheres were counted using a light microscope seven days after the suspension and collected for further study.

### Differentiation of piNSCs

To induce spontaneous neuronal differentiation, piNSCs (P20) were dissociated and re-plated into a 6-well dish or a 24-well plate with Matrigel-coated at a density of 2 × 10^4^ cells per cm^2^ in the neuronal differentiation medium (the piNSCM without bFGF and hEGF). The media was replaced every 2 days for 14 days. Phase-contrast image analysis was performed daily to monitor cell differentiation in each well. At days 0 (proliferating piNSCs) and 14 (neuronal differentiation), the cells were fixed with 4% paraformaldehyde for immunofluorescence analysis and manually detached for western blot analysis.

### Cell Proliferation

The cells were seeded at a density of 1 × 10^4^ cells per well in a 96-well plate and maintained for two days in an iNSCM. Cell proliferation was assessed using the Cell Counting Kit-8 (CCK-8) assay at various time points during culture (at 12, 24, 36, and 48 h). To evaluate cell proliferation, 10 μl of the CCK-8 reagent was applied to live cells in 96-well plates, and suspension NSCs (100 μl/well) were incubated for 3 h at 37°C with 5% CO_2_. The absorbance at 450 nm was used to determine the number of cells. The CCK8 standard curve was used to quantify the number of viable cells according to the Dojindo CCK-8 procedure. Briefly, cells were seeded in 96-well plates at 2.5 × 10^4^, 5 × 10^4^, 10 × 10^4^, 20 × 10^4^, 40 × 10^4^, and 80 × 10^4^ cells/well. The wells containing only media (without cells) were used to determine the background. Then, 10 μl of CCK8 was added to each well, and the plates were incubated at 37°C for 3 h. The CCK8 standard curve was established by plotting the number of cells vs. the absorbance.

### Immunofluorescence and Imaging Analysis

Immunofluorescence analysis was utilized to detect NSC markers and distinguish neuronal cell lineages. Samples were fixed with 4% paraformaldehyde in cold phosphate-buffered saline (PBS) at 37°C for 15 min. Then, samples were permeabilized with 0.25% Triton-X 100 in PBS at 37°C for 10 min and incubated with a non-specific binding blocking solution (2% bovine serum albumin in PBS) at 37°C for 1 h. Samples were treated overnight at 4°C in the dark with primary antibodies and then for 1 h at 37°C with secondary antibodies, as shown in Table 1. The coverslips were mounted on glass slides with an antifade mounting medium with DAPI (Vectashield, Vector Laboratories, Burlingame, CA, USA), and visualized using a Leica DMi8 inverted fluorescence microscope equipped with a Leica DFC7000 camera and the TCS SP8 confocal microscope equipped with a DFC3000G camera (Leica Microsystems, Wetzlar, Germany). For each sample, at least 40 z-stacks with 0.6–0.7 m intervals were acquired. All images were analyzed using the Leica Application Suite X (LAS X) imaging or ImageJ (NIH, USA) software to detect single fluorescence intensity measurements, the number of fluorescence positive cells, and co-localization. The images were measured in 20 randomly selected fields on each slide at a magnification of ×200. At least three slides were scanned for each group to determine the expression of these markers (*n* = 3 independent experiments). Data are presented as the mean fluorescence intensity value ± SEM after background signal subtraction. The percentage of positive cells per total number was based on the number of fluorescence marker-positive cells and DAPI-positive cell numbers measured by DAPI nuclear staining using ImageJ.

**Table 1:** List of antibodies used for immunofluorescence and western blot analysis.

**Markers**	**Antibodies**	**Dilution**	**Companies**	**Catalog number**
Cell proliferation	Rabbit anti-KI-67	1:100	Santa cruz	sc-15402
Pluripotency	Goat anti-OCT4	1:200	Santa cruz	sc-8628
	Goat anti-NANOG	1:100	R&D system	AF 1997
	Mouse anti-SSEA1	1:100	Santa cruz	sc-101462
Neural stem cells	Rabbit anti-PAX6	1:100	Thermo fisher scientific	PA5-85374
	Rabbit anti-SOX2	1:100	Santa cruz	sc-20088
	Mouse anti-NESTIN	1:100	Millipore	MAB5326
	Mouse anti-VIMENTIN	1:100	Santa cruz	sc-6260
Neural lineage	Rabbit anti-TUJ	1:100	Abcam	AB18207
	Mouse anti-MAP	1:100	Santa cruz	sc-32791
	Rabbit anti-GFAP	1:100	Millipore	AB5804
	Mouse anti-O4	1:100	Millipore	MAB5804
	Mouse anti-SYP	1:100	Abcam	AB8049
	Rabbit anti-TH	1:100	Abcam	AB137869
Housekeeping protein	Rabbit anti-β-actin	1:500	Cell signaling	4970s
Secondary antibodies	Alexa Fluor 488 donkey anti-rabbit IgG	1:1,000	Thermo fisher scientific	A-21206
	Alexa Fluor 488 donkey anti-mouse IgG	1:1,000	Abcam	Ab150105
	Alexa Fluor 488 donkey anti-goat IgG	1:1,000	Thermo fisher scientific	A11055
	Alexa Fluor 594 donkey anti-rabbit IgG	1:1,000	Thermo fisher scientific	A-21207
	Alexa Fluor 594 donkey anti-mouse IgG	1:1,000	Thermo fisher scientific	A-21203
	Anti-mouse IgM-PE	1:1,000	Santa cruz	sc-3768

### Western Blot Analysis

The cells were lysed using sonication in a radioimmunoprecipitation assay buffer, and the total protein concentration was examined by a protein assay kit (Bio-Rad Laboratory, Hercules, CA, USA). Western blot analysis was performed using the Jess^TM^ Simple Western system, an automated capillary-based size separation technique (ProteinSimple, San Jose, CA, USA) (39). The proteins of interest were separated and quantified using the 12-230-kDa and 66-440-kDa Jess separation modules (SM-W004 and SM-W008, ProteinSimple) according to the manufacturer's instructions. The primary and secondary antibodies used in these studies are listed in Table 1. The chemiluminescent detection was achieved using peroxide/luminol-S (ProteinSimple). The chemiluminescent images of separated proteins in the capillary were acquired with Compass Simple Western version 5.0.1 software (Build 0911, Protein Simple) that automatically measured the area (chemiluminescence intensity). The results are shown as electropherograms reflecting the peak of the chemiluminescence intensity and as a lane view from the chemiluminescence signal detected in the capillary. The relative density was calculated by dividing the peak area of the protein of interest by the peak area of β-actin.

### Karyotype Analysis

The karyotype analysis followed a previously described procedure with minor modifications (40). Briefly, piNSC lines (P20) were cultured in a 6 cm culture dish at approximately 70% confluence and treated with a 5 μg/mL Colcemid solution (KaryoMAX™ Solution) for 1 h at 37°C. The cells were gently dissociated using Versene® Solution and then treated for 15 min at 37°C with a hypotonic solution (75 M KCL). They were treated three times in a cold fixing solution (1:3 acetic acid to methanol concentration). The fixed cells were transferred to cool microscope slides and maintained at 37°C overnight. The slides were stained with Giemsa solution after soaking in a 0.05% trypsin EDTA solution at 37°C. The images of 50 metaphases were captured by a Nikon Eclipse Ni with DS-Ri2 camera (Nikon Instruments, Japan) and analyzed by LUCIA Cytogenetics (Nikon Instruments, Japan).

### SeV Genome and Transgenes Analysis

RT-PCR was used to evaluate the expression of the SeV genome and transgenes in both piNSCs. The RNeasy Mini Kit was used to lyse the cells and extract RNA (Genaid Biotech Ltd., New Taipei City, Taiwan). After that, 1 g of total RNA was reverse transcribed to cDNA using the SuperScript™ III First-Strand Synthesis System. In the PCR process, 50 ng template cDNA, 12.5L GoTaq PCR Master Mix (Promega, WI, USA), and 0.2 M of each primer were employed. PCR-amplified separation was achieved on 2% agarose gels and imaged using GelRed® nucleic acid staining (Biotium, Fremont, CA, USA). The relative expression levels of porcine endogenous genes of piNSC were also determined by RT-qPCR using the QuantiTect SYBR Green PCR Kit (QIAGEN, Germany). The expression levels of each mRNA were normalized to those of the pig β-actin gene, a housekeeping control. Each 20-μL PCR reaction consisted of 50-ng cDNA, 1× QuantiTect SYBR Green PCR master mix, and 0.2 μM of each primer. The samples were incubated at 95°C for 15 s, followed by 40 cycles of denaturation at 94°C for 15 s, annealing at 60°C for 15 s, and extension at 72°C for 30 s. The threshold cycle (C_T_) values of endogenous genes and β-actin were used for relative quantification using the comparative C_T_ (ΔΔC_T_) method. The real-time data were analyzed using DataAssist software version 3.01 (Applied biosystems, Life Technologies Corp., NY, USA). Table 2 lists the primer sequences.

**Table 2:** Primers used for reverse transcription polymerase chain reaction.

**Genes**	**Primer sequence (5** ^**′**^ **−3** ^**′**^ **)**		**Product size (bp)**
	**Forward**	**Reverse**	
**Exogenous genes**			
*SeV*	GGA TCA CTA GGT GAT ATC GAG C	ACC AGA CAA GAG TTT AAG AGA TAT GTA TC	181
*hOCT4*	CCC GAA AGA GAA AGC GAA CCA G	AAT GTA TCG AAG GTG CTC AA	483
*hSOX2*	ATG CAC CGC TAC GAC GTG AGC GC	AAT GTA TCG AAG GTG CTC AA	451
*hKLF4*	TTC CTG CAT GCC AGA GGA GCC C	AAT GTA TCG AAG GTG CTC AA	410
*hC-MYC*	TAA CTG ACT AGC AGG CTT GTC G	TCC ACA TAC AGT CCT GGA TGA TG	532
**Endogenous genes**			
*pOCT4*	ACA AGG AGA AGC TGG AGC CG	CGC GGA CCA CAT CCT TCT CT	752
*pSOX2*	GGT TAC CTC TTC CCA CTC CA	CAA AAA TAG TCC CCC CAA AAG	450
*pNANOG*	TCT GTG TCA GTT TGA GGG ACA GG	AAC AAG TAA AGC CTC CCT ATC CCA	120
**Oligodendrocyte gene**			
*pMBP*	GAG GCA GAG CTC CTG ACT ACA AA	GTC CCG TCC CAG CTT	101
**Housekeeping gene**			
*β-actin*	CGG GAC CTG ACT GAC TAC CTC	CCT TAA TGT CAC GCA CGA TTT CC	93

### Statistical Analysis

Each experiment was conducted a minimum of three times. The quantitative results were expressed as the mean ± standard error of the mean (SEM). The data was analyzed statistically using one-way analysis of variance for comparisons of more than two groups and the student's *t*-test for comparisons of the two groups. Additionally, Tukey's test was employed as a post *hoc* multiple comparison test for differences. All statistical analyses were carried out using SPSS version 25 software (IBM, USA). Statistical significance was defined as *p* < 0.05.

## Results

### piNSC Lines Are Generated From PTFs Using the Non-integrative Sendai Virus

To address the question of whether PTFs can be directly converted into stably expanding multipotent piNSCs, PTFs were isolated from the tail of a three-day-old, crossbred piglet (Large White/Landrace × Duroc). Non-integrative Sendai viral vectors carrying OSKM were transfected into PTFs for 24 h (Figure 1B). After that, the cells were cultured in FM for one day to recover and then in iNSCM for another day. On day 7 after transduction, the cells were dissociated and transferred onto Matrigel-coated 6-well plates in piNSCM at a density of 1 × 10^4^ cells per well. On day 11 after transduction, iNSC clusters with neuroepithelial-like morphology emerged and developed quickly over the next week, revealing unclear and irregular boundaries with filament-like cells extending outward, which had a distinct morphology from that of porcine iPSC (piPSC) colonies. On days 16–21, the morphologically neuroepithelial-like colonies were mechanically triturated into small clusters and re-plated onto Matrigel-coated coverslips for immunostaining or onto a Matrigel-coated one-well dish for cell expansion in iNSCM (Figures 1C,D). The neuroepithelial colonies expressing early NSC markers such as PAX6, NESTIN, and SOX2 were continuously propagated using TrypLE Select Enzyme along the serial passaging for further characterization (Figures 1E–G). Hence, the transfection efficiency was 0.40%, as measured by the number of neuroepithelial colonies expressing PAX6, NESTIN, and SOX2 divided by the total number of transfected cells. Additionally, immunofluorescence analysis revealed that the piNSC-like colonies (passage 2) were negative for the pluripotency markers OCT4 and NANOG, indicating that the colonies were not in a pluripotent state (Supplementary Figure 1). We generated a total of nine iNSC lines capable of proliferation in adherent monolayers (2D) (Figure 1H) or neurospheres (3D) (Figure 1I) as suspension-grown neural cell aggregates and differentiation into neural lineages (Figure 1J). We used a pig tail to generate PTFs and iNSCs in this investigation. The generation of piNSCs was established in three replicates. We chose only two iNSC lines, namely VSMUi002-B and VSMUi002-E, based on their indefinite self-renewal potential and multipotency differentiation for further analysis. Each experiment involving two piNSC lines was performed at least three times.

### piNSC Lines Exhibit Characteristics of NSCs

**Figure 2:**
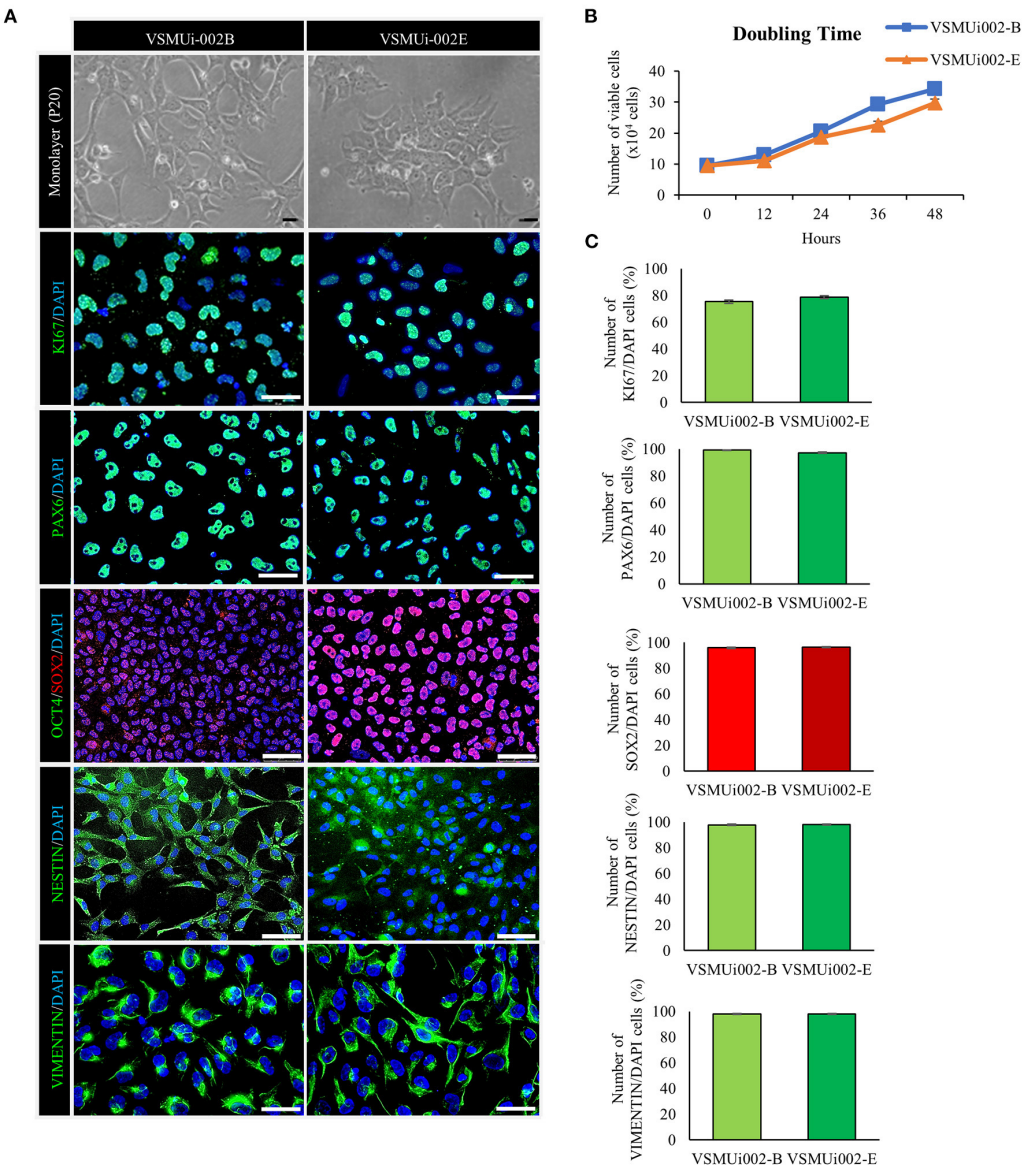
Characterization of porcine induced neural stem cell (piNSC) lines. **(A)** At passage 20 (P20), the piNSCs had a neuroepithelial morphology and expressed the cell proliferation marker KI67 (green) as well as neural stem cell markers including PAX6 (green), SOX2 (red), NESTIN (green), and VIMENTIN (green), but not the pluripotency marker OCT4 (green). DAPI staining is blue. **(B)** The population cell doubling time of piNSC lines. **(C)** Quantitative evaluation of cell proliferation and neural stem cell markers in piNSC lines. Means with different lowercase letters are significantly different at *P* < 0.05. Scale bars in **(A)** represent 10 μm.

**Figure 3:**
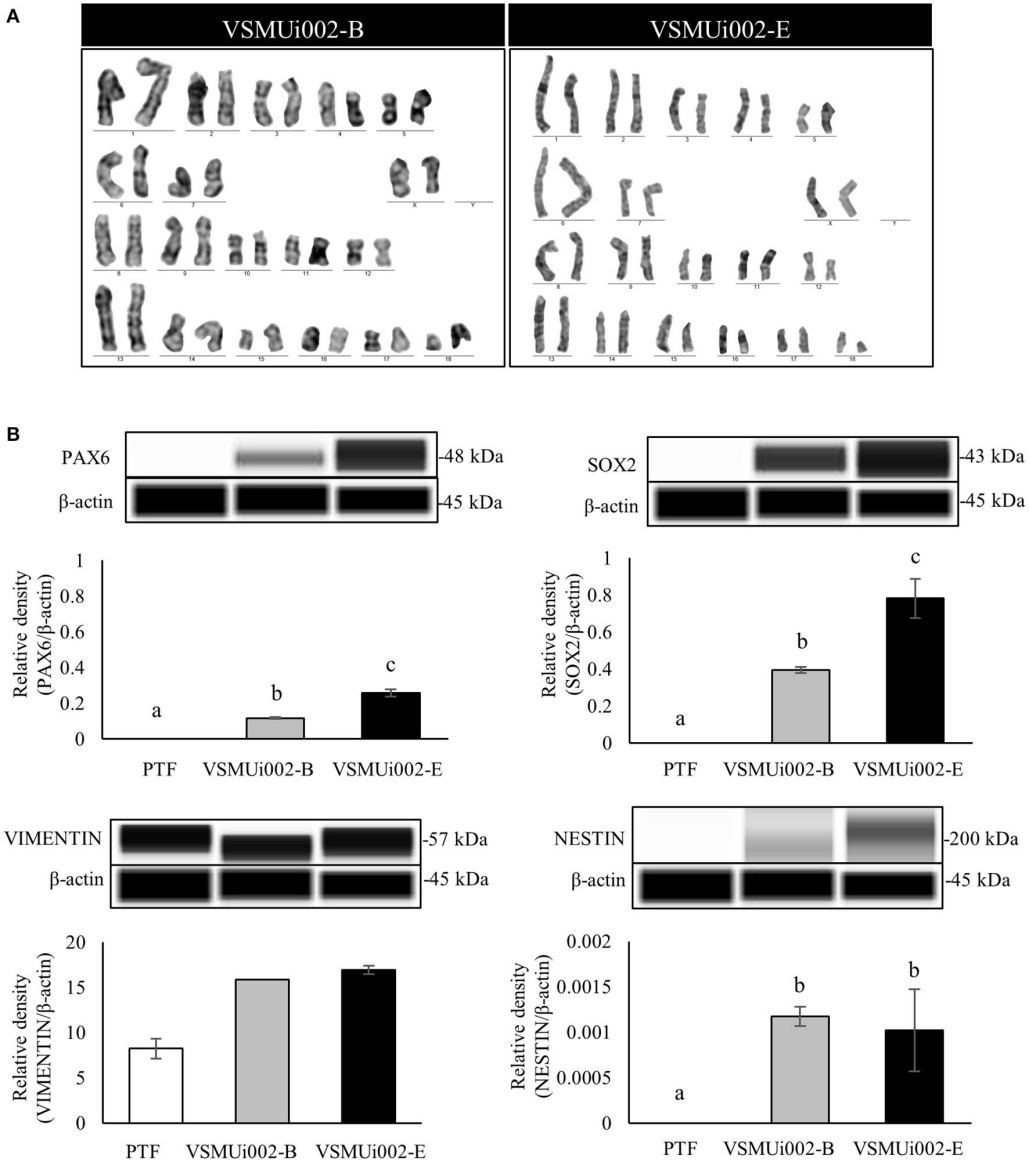
Characterization of porcine induced neural stem cell (piNSC) lines (continuous). **(A)** Karyotype analysis of the piNSC lines showed a normal karyotype. **(B)** Western blot images of neural stem cell expressions (PAX6, SOX2, VIMENTIN, and NESTIN) and quantification of the western blot results. β-actin was used as an internal control. Means with different lowercase letters are significantly different at *P* < 0.05.

**Figure 4:**
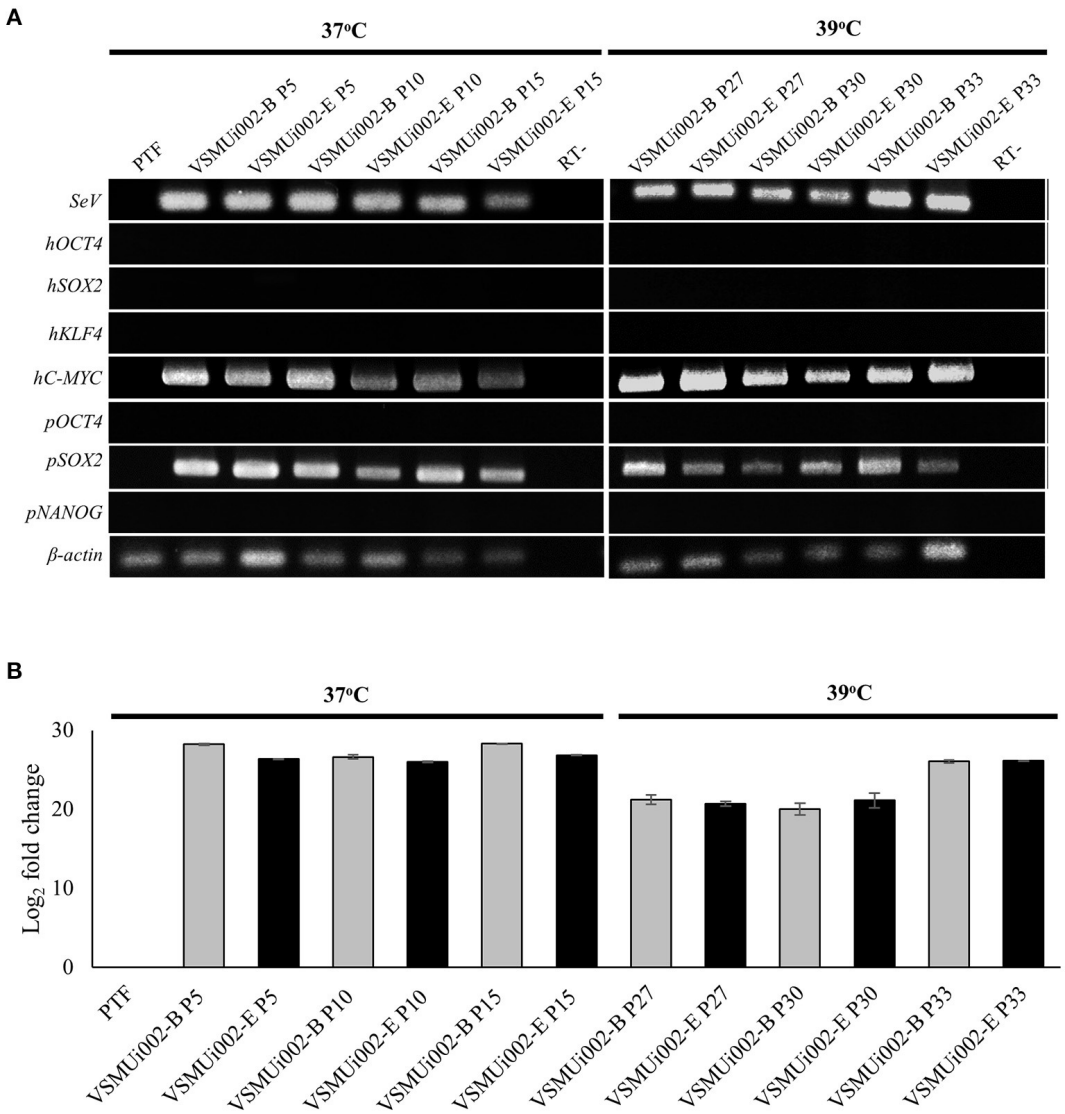
Expression of exogenous and endogenous factors. **(A)** Expression of exogenous factors (*hOCT4, hSOX2, hKLF4, hMYC*), Sendai virus (SeV) RNA and endogenous factors (*pOCT4, pSOX2*, and *pNANOG*) was analyzed between 37 and 39°C for viral inactivation. **(B)** Fold change of SeV RNA expression in PTF and piNSCs at 37 and 39°C as determined by real time-PCR.

At passage 20, the piNSC lines (VSMUi002-B and VSMUi002-E) displayed a neuroepithelial morphology in adherent monolayers (Figure 2A). The population cell doubling time of the VSMUi002-B and VSMUi002-E cell lines was approximately 24 h, with no significant difference (*P* > 0.05) (Figure 2B). Both piNSC lines showed good proliferative capacity, as indicated by the high percentage of cells expressing KI67 (75.3 ± 1.18% and 78.6 ± 1.19%) (Figures 2A,C), and had been passaged more than 40 times. Hence, both iNSC lines had a strong capacity for self-renewal. They exhibited a typical diploid porcine karyotype (38, XX) during long-term culture (Figure 3A). Moreover, they displayed a high percentage of cells expressing the NSC markers, with nearly 100% of cells staining positive for PAX6, SOX2, NESTIN, and VIMENTIN, as determined by quantitative immunofluorescence analysis, which indicated the formation of a highly homogeneous population (Figures 2A,C). To corroborate the immunofluorescence results, endogenous PAX6, SOX2, VIMENTIN, and NESTIN proteins in piNSCs were quantified by western blot analysis in comparison with their parental PTFs. The expressions of PAX6 and SOX2 were substantially higher in VSMUi002-E than in VSMUi002-B. NESTIN expression was significantly higher in both piNSC lines than in PTFs. The PTFs lacked PAX6, SOX2, and NESTIN protein expression (Figure 3B; Supplementary Figure 3). On the other hand, the expression of VIMENTIN was higher in both piNSC lines than in PTFs (Figure 3B; Supplementary Figure 3). To investigate the pluripotent state, the piNSC lines (VSMUi002-B and VSMUi002-E) were analyzed using RT-PCR and immunofluorescence staining for pluripotency markers. RT-PCR analysis revealed that both piNSC lines were negative for pluripotency genes (*pOCT4* and *pNANOG*) during early (P5, P10, and P15) and late passages (P27, P30, and P33) (Figure 4A; Supplementary Figures 4, 5). Immunofluorescence staining revealed that both piNSC lines at passage 20 did not express pluripotency-related markers, including OCT4, NANOG, and SSEA1 (Figure 2A; Supplementary Figure 2). Hence, the piNSCs were not in a pluripotent state due to the absence of pluripotency markers. The piPSC line (VSMUi001-A) was used as a positive control to test for pluripotency markers (Supplementary Figure 2). To eliminate temperature-sensitive SeV vectors, piNSCs at passage 15 were grown at 39°C for viral inactivation, and cells were collected weekly for PCR detection of the residual virus. After 5 weeks of continuous culture, the residual virus was positive at passage 33, which indicated that SeV vectors were difficult to eliminate entirely. We also excluded the irregular integration of SeV by comparing the amount of SeV in PTFs and piNSCs using real-time RT-PCR, and found that the SeV expression was significantly decreased at 39°C (after the temperature change) compared to that at 37°C (Figure 4B). This finding implies that SeV did not integrate into the host genome. Moreover, piNSCs had undetectable levels of the three transgenes (*hOCT4, hSOX2*, and *hKLF4*), regardless of the temperature treatment, as determined by PCR. On the other hand, piNSCs remained positive for *hc-MYC*, regardless of the temperature treatment (Figure 4A).

**Figure 5:**
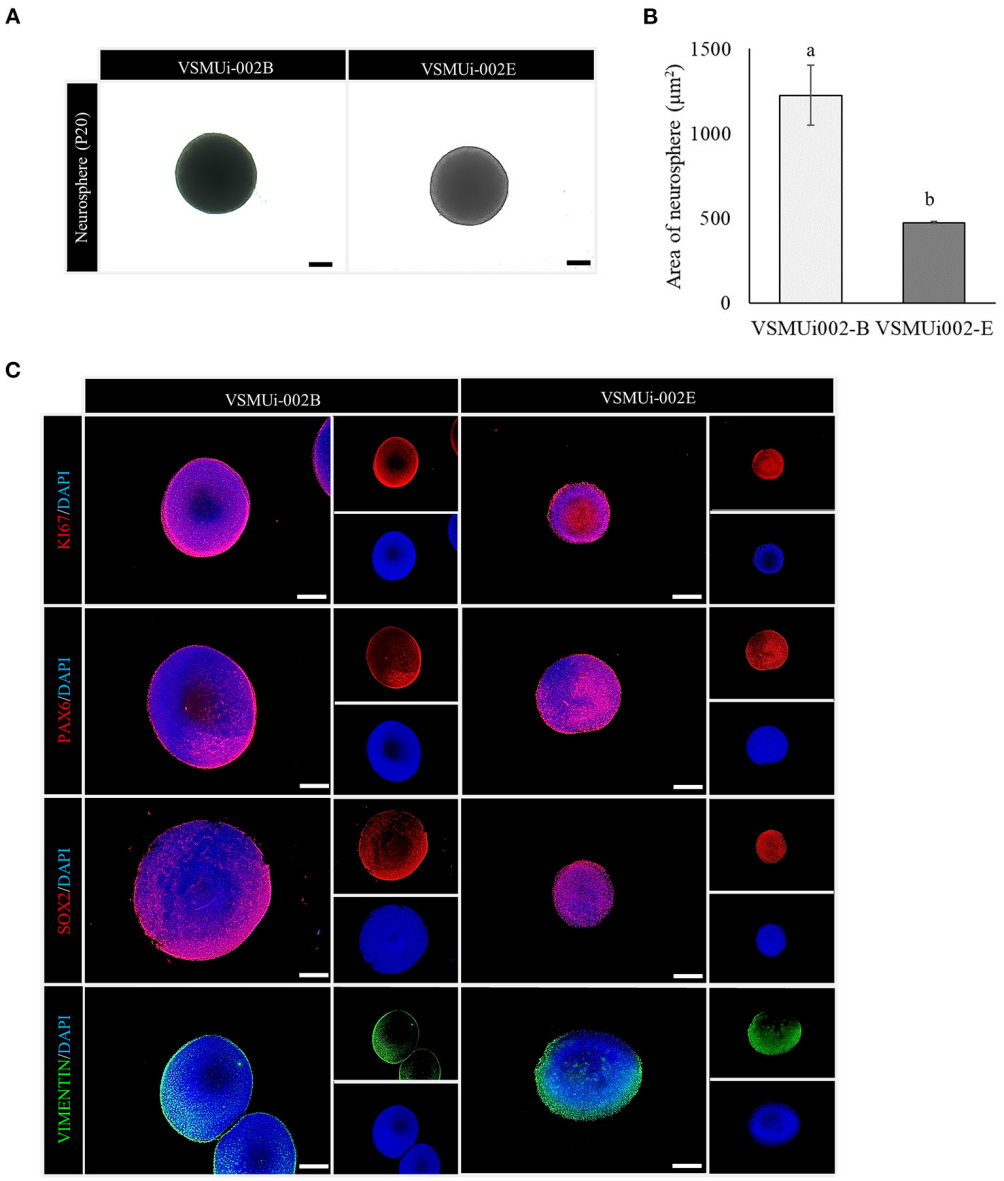
Neurosphere formation of porcine induced neural stem cell (piNSC) lines. **(A)** Phase-contrast image of neurospheres on day 7. **(B)** Average area of neurospheres. Means with different lowercase letters are significantly different at *P* < 0.05. **(C)** Immunofluorescence staining of neurospheres exhibits the expression of the cell proliferation marker KI67 (red) as well as neural stem cell markers, including PAX6 (red), SOX2 (red), and VIMENTIN (green). The scale bars in **(A)** and **(C)** represent 100 μm.

Both piNSC lines were able to form neurospheres (3D) in suspension cultures with similar efficiency, which were homogeneous in size and shape on day 7 (Figures 5A,B). Additionally, immunofluorescence labeling revealed that the neurospheres expressed NSC markers (PAX6, SOX2, VIMENTIN) and a proliferation marker (KI67) (Figure 5C). Thus, piNSCs display neural progenitor features that can be obtained from the PTFs by SeV reprogramming.

### The piNSCs Spontaneously Differentiate to Neurons and Glia

**Figure 6:**
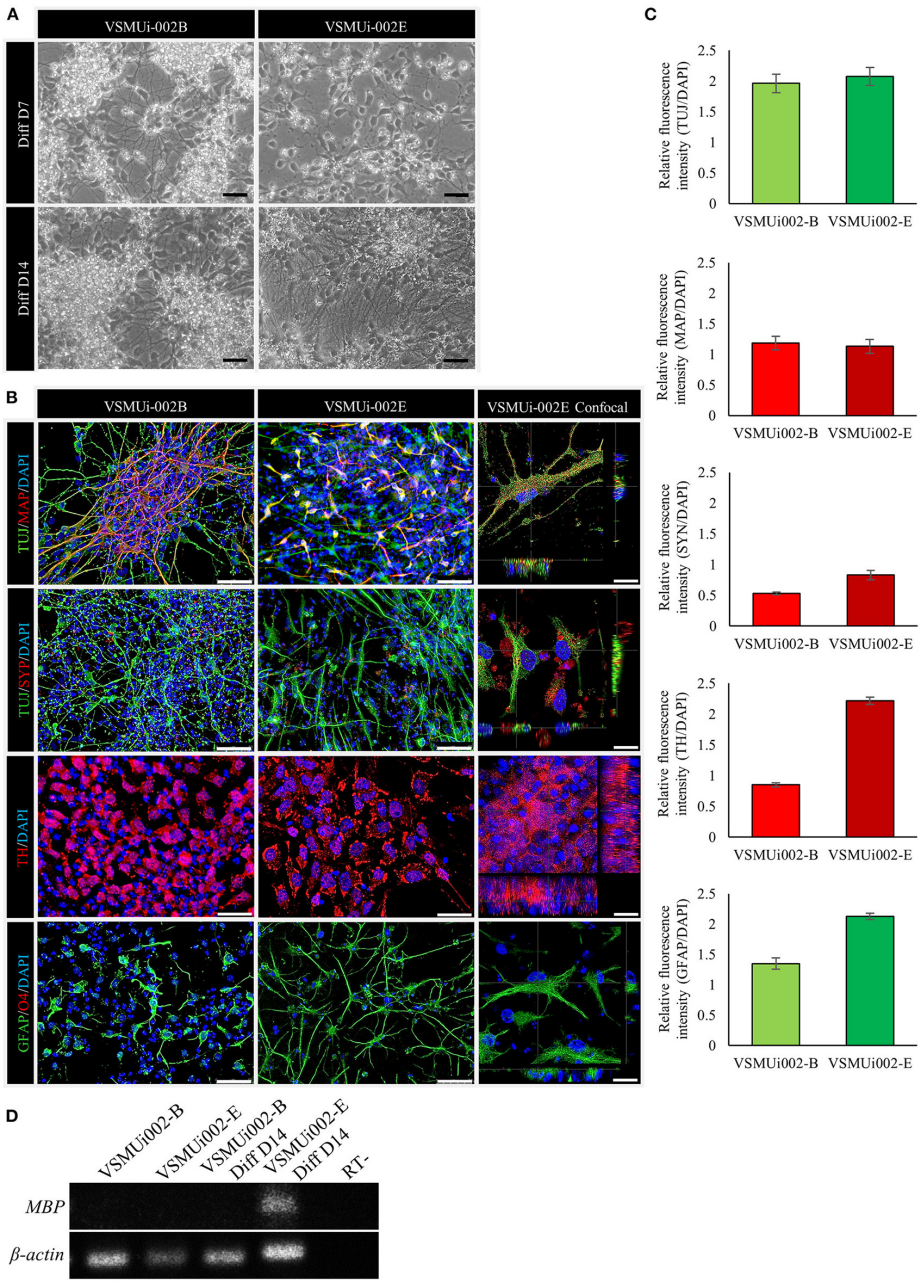
*In vitro* differentiation potential of porcine induced neural stem cell (piNSC) lines. **(A)** Phase-contrast image of neural differentiation derived from piNSC lines on day 7 and day 14 of differentiation. **(B)** Neural differentiated from piNSC lines expressed immature neuronal marker (TUJ1, green), mature neuronal marker (MAP2, red), synaptic protein synaptophysin (SYP, red), dopamine-secreting neurons (TH, red), and astrocyte (GFAP, green). DAPI staining shown in blue. **(C)** Quantitative analysis of neurons and astrocytes derived from piNSC lines. The mean fluorescence signals for TUJ1, MAP2, SYP, TH, and GFAP were measured in 20 images per marker in each cell line under identical optical settings. **(D)** The VSMUi002-E piNSC line exhibited myelin basic protein (MBP), which is predominantly expressed in oligodendrocytes. The scale bars in **(A)** and **(B)** represent 20 and 50 μm, respectively.

**Figure 7:**
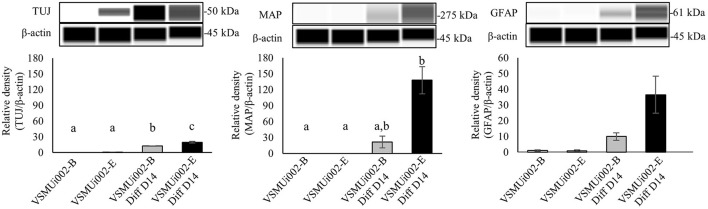
Western blot analysis of neural differentiation derived from porcine induced neural stem cell (piNSC) lines. Western blot images of immature neuronal (TUJ1), mature neuronal (MAP2) and astrocyte (GFAP) expression and quantification of the western blot results. β-actin was used as an internal control. Means with different lowercase letters are significantly different at *P* < 0.05.

To determine the ability of piNSCs to differentiate spontaneously, they were dissociated into single cells and grown on a Matrigel substrate in a neural differentiation medium. After seven days of differentiation, piNSCs revealed significant morphological alterations, including a decrease in cell body size (Figure 6A), and expressed the immature neuronal marker (TUJ1) (Figure 6B). At day 14, the piNSCs exhibited mature neuronal morphology, including extensive and complex neurites (Figure 6A), which were positive for the mature neuronal marker (MAP2) (Figure 6B). The merged immunofluorescence images revealed a co-localization of TUJ and MAP. Consistent with these findings, high levels of MAP2 protein expression colocalized with TUJ1 staining in VSMUi002-B and VSMUi002-E, with Pearson's correlation of 0.61 ± 0.02 and 0.79 ± 0.01, respectively. Additionally, some TUJ1-positive neurons were labeled with the synaptic protein synaptophysin (SYP) along neurites in a punctate manner (Figure 6B), which indicated possible synaptic connections. Furthermore, the piNSCs exhibited a high capacity for differentiation into dopamine-secreting neurons expressing tyrosine hydroxylase (TH) (Figure 6B). The piNSCs also developed into glial fibrillary acidic protein (GFAP)-positive astrocytes (Figure 6B). The staining intensities of all neuronal-positive cells (TUJ1, MAP2, TH, SYN, and GFAP) were the same in both iNSC lines (Figure 6C). Moreover, the RT-PCR analysis indicated an increase in myelin basic protein (MBP) expression, which is expressed mainly in oligodendrocytes (Figure 6D; Supplementary Figure 6), thus indicating the existence of oligodendrocytes in differentiated derivatives. To corroborate the immunofluorescence results, endogenous TUJ1, MAP2, and GFAP proteins in iNSC-derived neural differentiation were quantified by western blot analysis in comparison with their parental iNSCs. The expression of these proteins was significantly higher in neuronal differentiation from VSMUi002-E than from VSMUi002-B (Figure 7; Supplementary Figure 7). Both iNSCs lacked MAP2 and GFAP protein expressions (Figure 7; Supplementary Figure 7). Interestingly, VSMUi002-E also expressed TUJ1 during the NSC stage (Figure 7; Supplementary Figure 7). Considered together, our data indicate that piNSCs have the capacity for multipotent neuronal differentiation.

## Discussion

Here, in comparison with prior studies, we explain several benefits of utilizing the temperature-sensitive SeV approach for reprogramming porcine fibroblasts into iNSCs: (1) Our piNSCs exhibit self-renewal and multipotency into neuronal and glial lineages without going through a pluripotent state, making them a safer alternative to piPSCs and (2) Stable and self-renewing iNSCs can be generated from pig sources, which is the ultimate cell source for studying pig disease in veterinary medicine and may be advantageous for determining the efficacy of iNSCs prior to human trials (29).

The integration of techniques is frequently based on viral vector systems, which have been widely utilized and verified for the production of transgenic animal models and cell lines. Retroviral and lentiviral vectors are incorporated into the target genome, causing lasting genetic changes to the host genome and frequently affecting the transcriptome through persistent transgene expression (9, 13, 15, 30). Thus, while numerous studies have effectively transdifferentiated somatic cells into iNSCs using retro- or lentiviral vectors, a transgenic factor-free procedure is desirable to minimize the possibility of irreversible genetic alterations impairing the normal function of generated iNSCs. Alternatively, non-integrating methods for generating transgene-free iNSCs were devised, including the use of episomal vectors and the SeV in combination with or without small molecules (19, 41, 42). The use of an episomal vector is inexpensive and easily adaptable for reprogramming a variety of cell types, including iPSCs and iNSCs. Episomal vectors are eliminated from these iPSCs via several passages through cell divisions, which can take several months to achieve transgene-free iPSCs (30, 32). However, Xu and colleagues indicated that piNPCs contained virtually undetectable amounts of EBNA-1, but episomal vector-transfected PFFs carried approximately 100 copies per cell, suggesting that EBNA-1 was not present in the piNPC genome (29). The SeV is a negative-sense single-stranded RNA virus that infects predominantly mammalian cells and replicates solely in their cytoplasm. In recent years, SeV-based vectors have increasingly been utilized to generate transgene-free iPSCs and have been modified to generate iNSCs from human and monkey postnatal and adult fibroblasts (18, 43). In this study, piNSC lines were successfully produced for the first time utilizing an integration-free SeV method, with greater reprograming efficacy (0.4%) than an integration-free episomal approach (0.002%) (29). In this study, we employed a commercially available reprogramming product, the temperature-sensitive SeV vector, which was designed to prevent replication at high temperatures, which results in the genome being passively eliminated during cell passage. The temperature-sensitive SeV vector is routinely used for generating human integration-free iNSC lines. Our findings indicated that the SeV vector and *hc-MYC* were expressed in piNSCs despite the high temperature-shift treatments that were utilized. The piNSC lines did not express *hOCT4, hSOX2*, and *hKLF4*, regardless of the temperature treatment. As previously described, the SeV vector is expressed and retained in the cytoplasm of piPSCs. The reason for the persistent expression of SeV and *hC-MYC* in our investigation is unclear. According to the Reprogramming Kit handbook, this might be because some cell strains (in this case, some species) take longer time to eliminate SeV and C-MYC vectors and become vector-free. Similar to Congras and colleagues, they generated piPSCs using the SeV vector and revealed that while *hOCT4* and *hMYC* expression were maintained throughout passages, *hSOX2* and *hKLF4* expression were undetectable following high temperature-shift treatments using RT-PCR. They demonstrated that exogenous factors are retained by the persistence and replication of the SeV genome in the cytoplasm of piPSCs rather than via genomic insertion (44). Similar to our result, SeV expression was significantly decreased after the temperature change.

Other integration-free systems rely on the usage of non-integrating lentiviral vectors (integrase-defective lentiviral vectors), Protein, mRNAs and chemical-based. For a non-integrating lentiviral vector system, a doxycycline-inducible lentivirus vector was recently modified to incorporate a loxP-flanked expression cassette, enabling the creation of iNSCs. This allowed for later Cre-mediated transgene elimination following iNSC production. This lentivirus vector system can directly convert human adult monocytes, dermal, and fetal pancreatic fibroblasts into transgene-free iNSCs (45). TAT-SOX2 recombinant protein transduction combined with a cocktail of small chemical compounds to convert human adult fibroblasts directly into genetic material-free iNPCs (46). Additionally, mRNAs-encoding SOX2 reprogrammed human mesenchymal stem cells into integration-free iNSCs (47). Although the mRNA-based reprogramming method demonstrated a reduced risk of insertional mutagenesis of the host genome, this method has several limitations, including poor stability and being time-consuming (48). Direct conversion by chemical reprogramming has been shown to be up to 30% efficient. For instance, the production of iNSCs from mouse fibroblasts and human urine cells utilized a cocktail of three small chemicals: valproic acid (a histone deacetylase inhibitor), CHIR99021 (a GSK3 inhibitor), and RepSox (a TGFb inhibitor). Other research used a mixture of different pathway modulators to induce somatic cells to convert into iNSCs, including A-83-01 (a TGFb inhibitor), purmorphamine (a sonic hedgehog agonist), and thiazovivin (a ROCK inhibitor) (49, 50). Conversely, distinct cell populations were sensitive to pharmacologically active compounds that modulate cellular pathways involved in epigenetic status, transcriptional changes, cell signaling, and metabolism in order for chemical reprogramming to occur (51). The important limitation for chemical reprogramming is the risk of chemical-induced cell toxicity and genotoxicity as unintended side effects, which must be considered for each substance to design a reliable and robust system for direct conversion (52).

Our piNSC lines exhibited NSC markers (PAX6, SOX2, NESTIN, and VIMENTIN) and demonstrated self-renewal potential as determined by immunocytochemical labeling for proliferation markers KI67 in conjunction with essential markers for NSCs, which were similar to previous studies (18, 29, 43). The SeV approach did not produce piPSC colonies with a high nucleus-to-cytoplasm ratio or clear colony borders, as previously described of piPSCs produced by viral vector-mediated methods (26, 53, 54). Furthermore, the lack of OCT4, NANOG, and SSEA1 demonstrated that piNSC lines were not in a pluripotent state. As a result, piNSCs are considered to be safer than piPSCs (15, 16). Hence, the SeV provides an alternative reprogramming method that generates iNSCs without going through a pluripotent state. Our piNSCs are multipotent stem cells that are capable of generating neurons and glial cells, similar to previous findings (29, 55). However, the differentiation capacity varies according to the iNSC population, and the differentiation technique is utilized *in vitro* (e.g., spontaneous, undirected, and directed *in vitro* differentiation approaches) (45). Our findings confirmed that these piNSCs are more prone to developing into neurons and astroglia than into oligodendrocytes, since development into oligodendrocytes was less effective due to the short differentiation phase, which was consistent with a previous study (13).

## Conclusions

Using the non-integration SeV for reprogramming, we effectively produced piNSC lines without going through an intermediate pluripotent stage. To our knowledge, this is the first attempt to use the SeV to directly reprogram somatic cells into NSCs in a porcine species. These piNSCs may provide an intriguing tool for determining the ultimate feasibility of disease modeling in pigs and cell-based regenerative treatment for human medicine. As a result, this finding is useful for veterinary medicine and the prospect of human medical translation.

## Data Availability Statement

The original contributions presented in the study are included in the article/Supplementary Material, further inquiries can be directed to the corresponding author.

## Ethics Statement

The animal study was reviewed and approved by the Institutional Animal Care and Use Committee at Faculty of Veterinary Science, Mahidol University, Thailand (Approval ID: MUVS-2015-49).

## Author Contributions

WC and SR were responsible for funding acquisition, conceptualization, original draft manuscript writing, execution of the majority of experiments, including the establishment and characterization of piNSCs. RR was responsible for the initial cell reprogramming process. LS was responsible for the molecular analysis. PJ, NC, and SC conducted cell culture, immunofluorescence, and imaging analyses. WC analyzed the data. JF and SR contributed to the review and editing of the final manuscript. All authors approved the final version submitted for publication.

## Funding

This research has received funding support from the NSRF via the Program Management Unit for Human Resources & Institutional Development, Research and Innovation [Grant number B05F630046] and Mahidol University [Basic Research Fund: fiscal year 2021].

## Conflict of Interest

The authors declare that the research was conducted in the absence of any commercial or financial relationships that could be construed as a potential conflict of interest.

## Publisher's Note

All claims expressed in this article are solely those of the authors and do not necessarily represent those of their affiliated organizations, or those of the publisher, the editors and the reviewers. Any product that may be evaluated in this article, or claim that may be made by its manufacturer, is not guaranteed or endorsed by the publisher.
