# Supplementary material for: Generation of Porcine Induced Neural Stem Cells Using the Sendai Virus
Source: Front Vet Sci. 2022 Jan 12;8:806785. doi: 10.3389/fvets.2021.806785 (PMC8790232; doi:10.3389/fvets.2021.806785)
Supplement: Supplementary file 1 [file Data_Sheet_1.PDF]

Supplementary Materials

1. Supplementary Figures

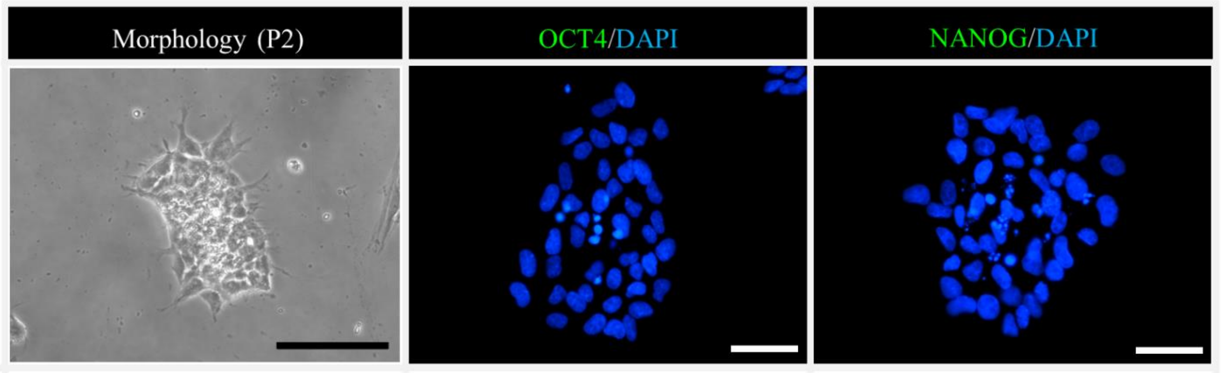

Supplementary Figure 1. The piNSCs at passage 2 (P2) exhibited unclear and irregular boundaries with filament-like cells extending outward and did not express pluripotency markers (OCT4 and NANOG). Scale bars represent 50 µm.

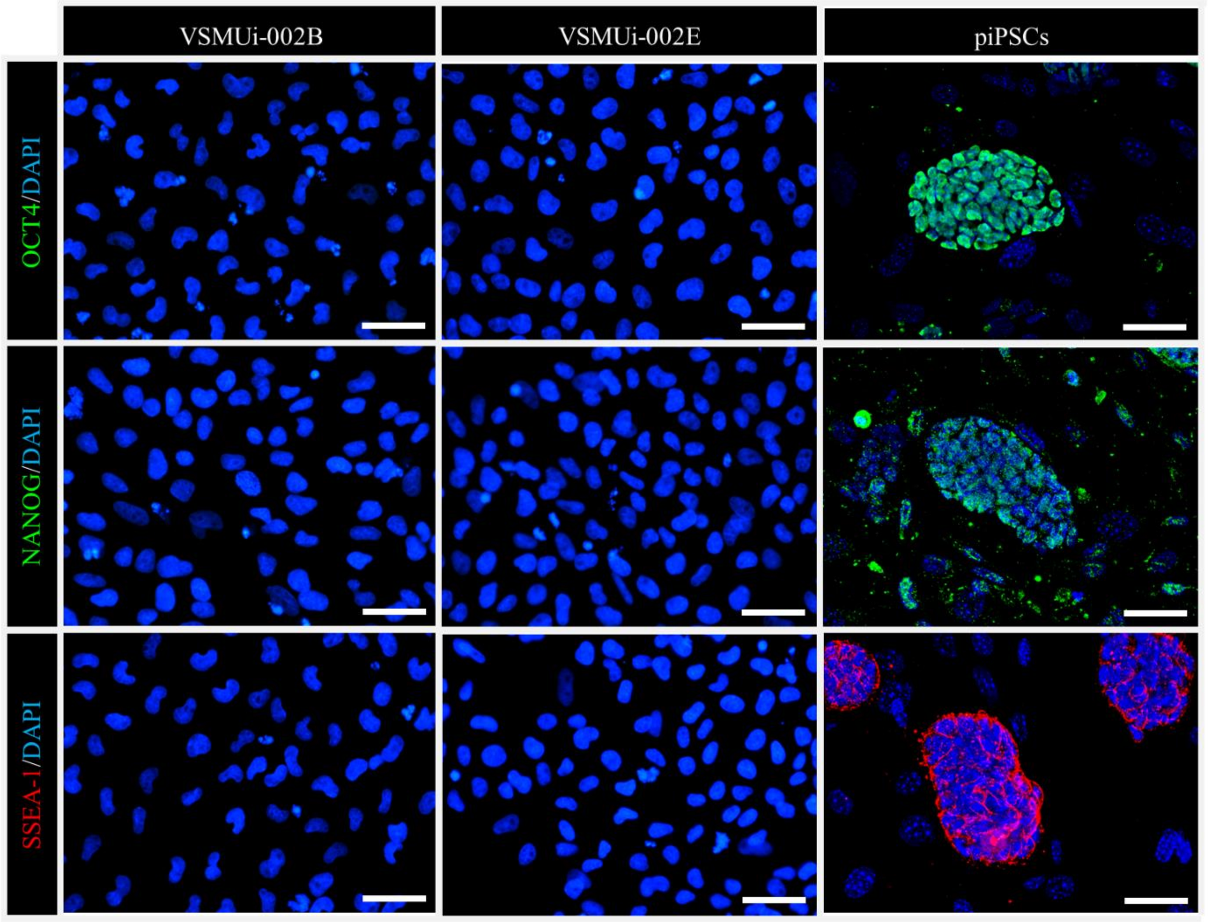

Supplementary Figure 2. At passage 20, the piNSCs did not express pluripotency markers (OCT4, NANOG, and SSEA1). As a positive control for pluripotency markers, the piPSC line (VSMUi001-A) was shown to be positive. Scale bars represent 50 µm.

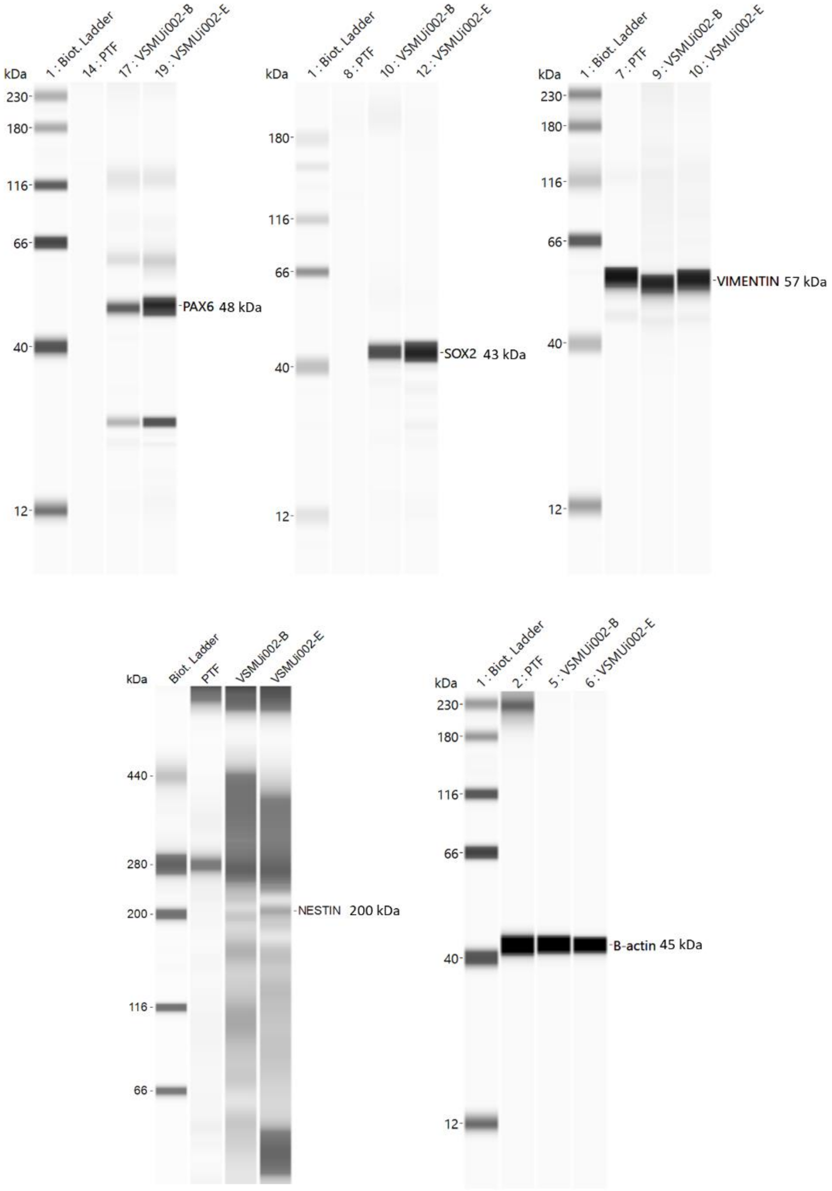

Supplementary Figure 3. Original blots of Western blot of Figure 3B

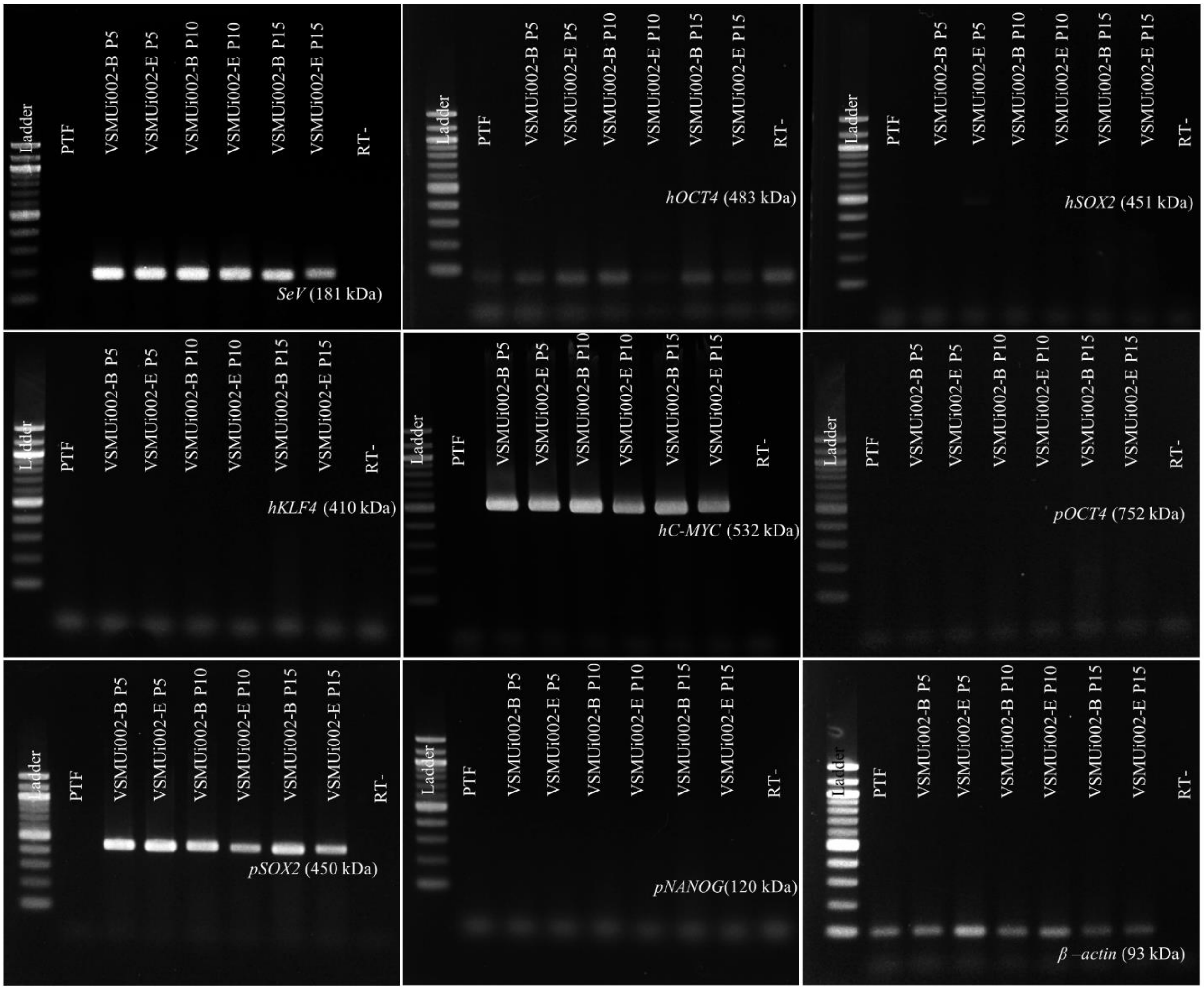

Supplementary Figure 4. Original RT-PCR of Figure 4A (37°C)

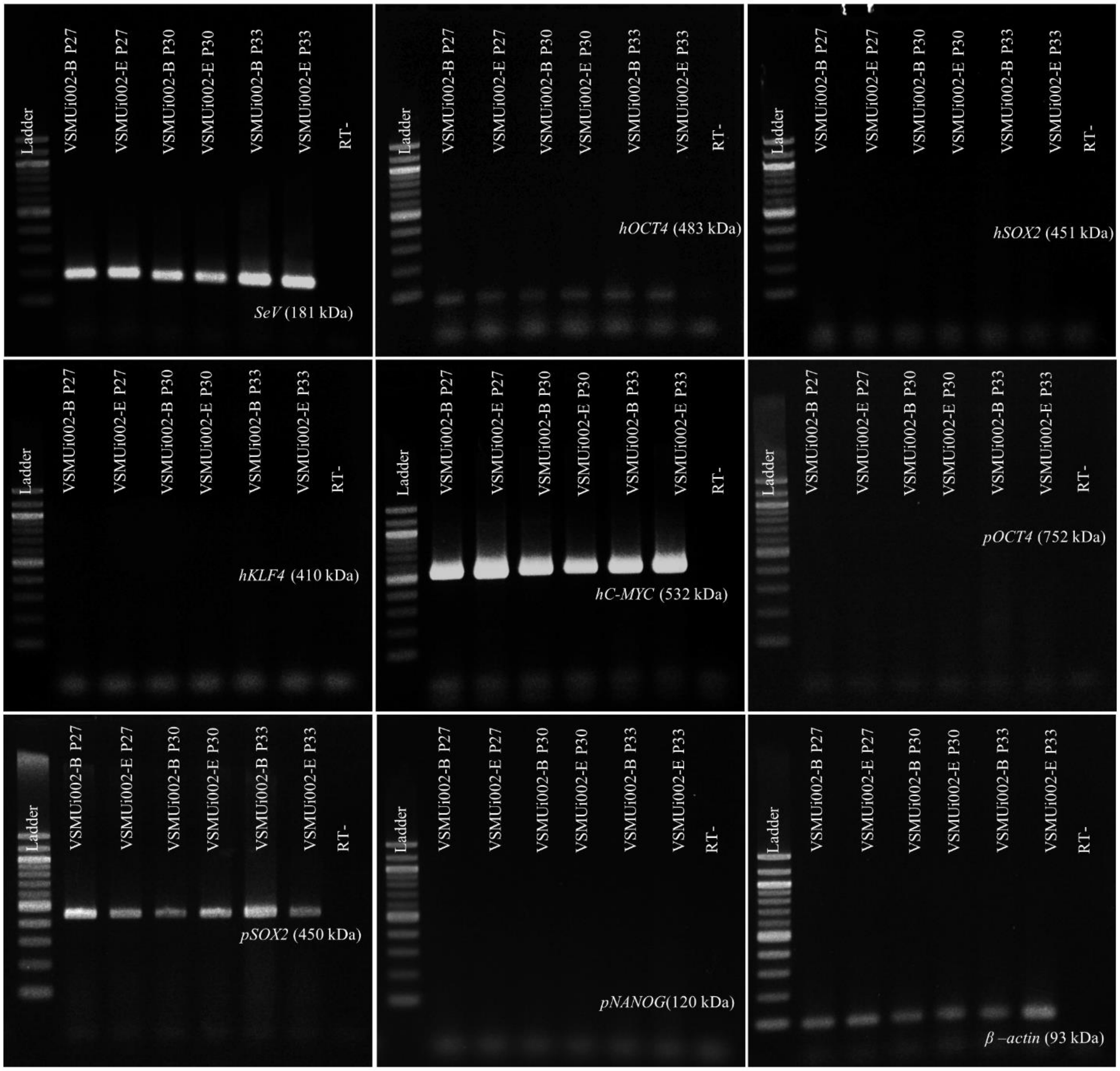

Supplementary Figure 5. Original RT-PCR of Figure 4A (39ºC)

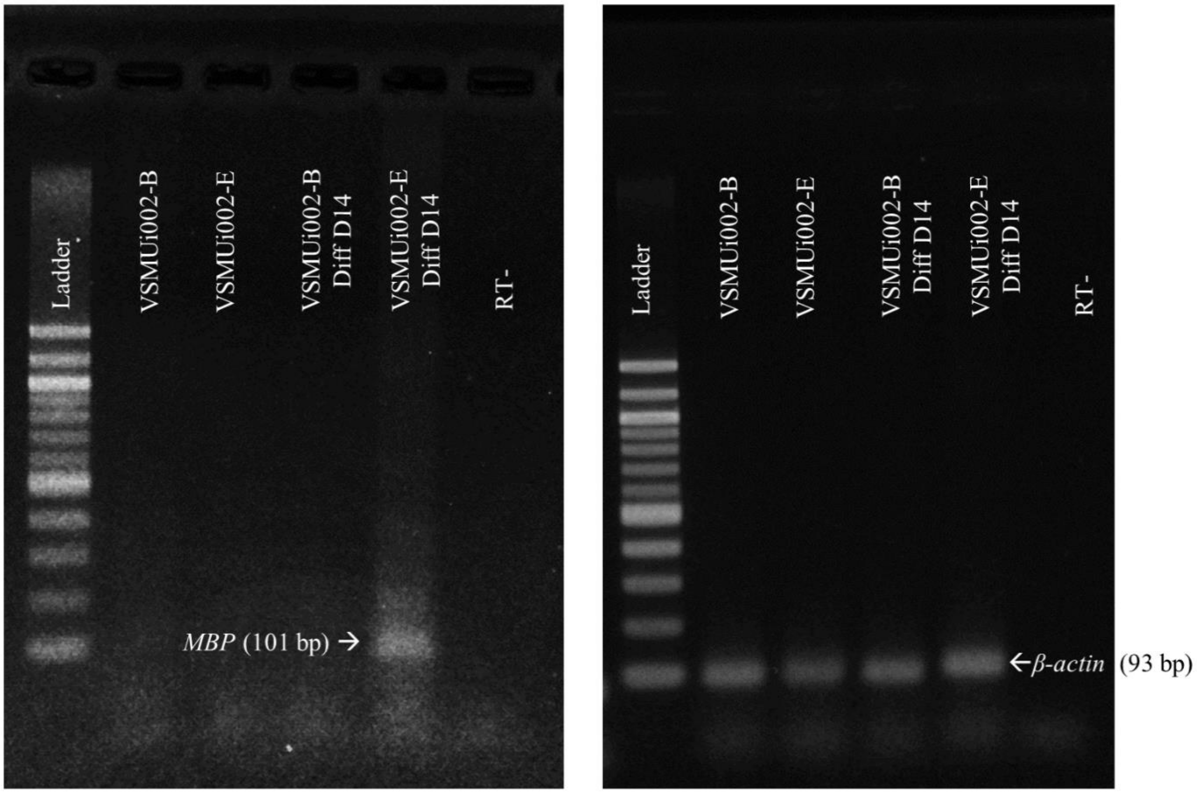

Supplementary Figure 6. Original RT-PCR of Figure 6D

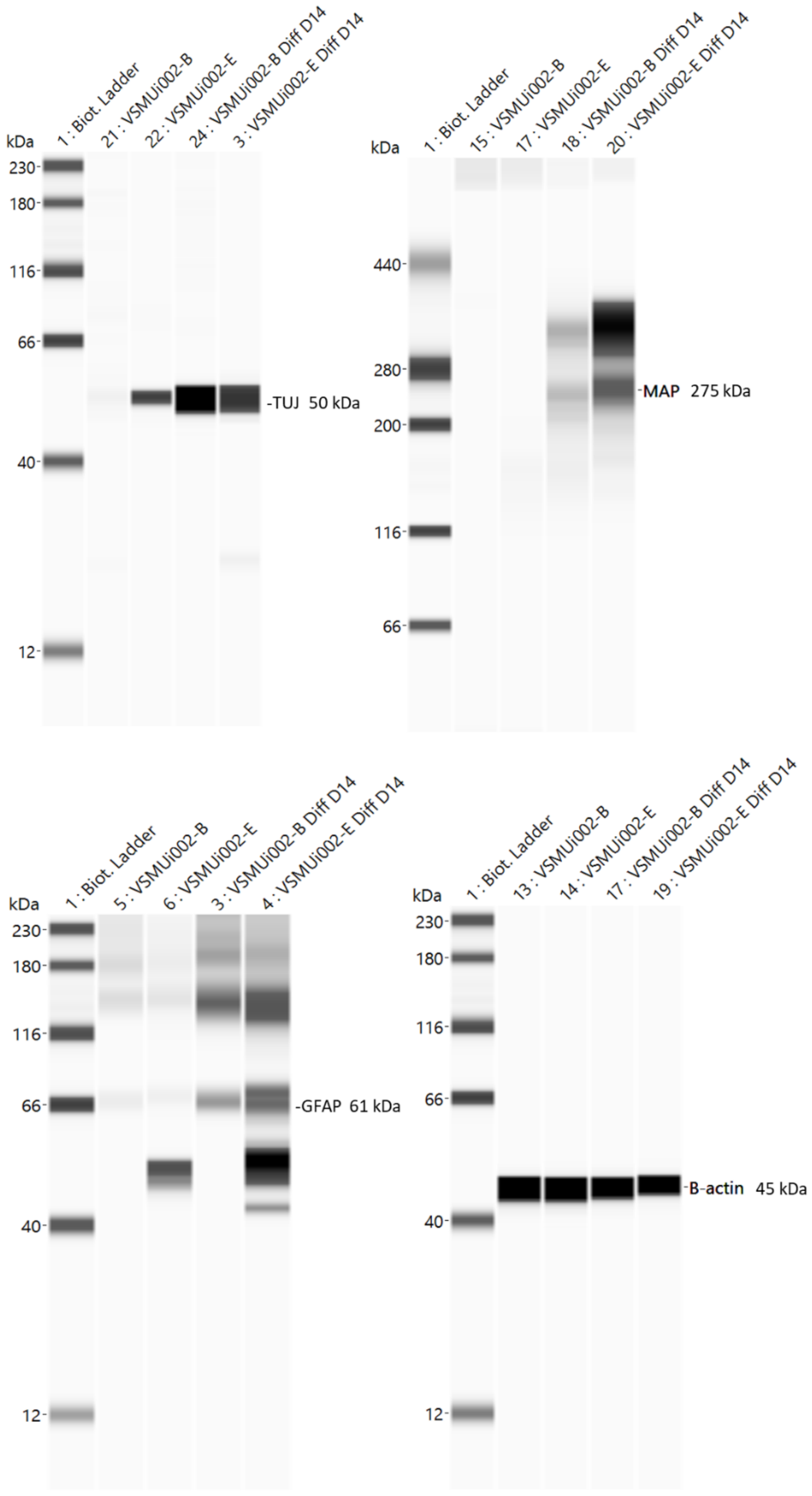

Supplementary Figure 7. Original blots of Western blot of Figure 6
